# UW Imaging of Seismic-Physical-Models in Air Using Fiber-Optic Fabry-Perot Interferometer

**DOI:** 10.3390/s17020397

**Published:** 2017-02-17

**Authors:** Qiangzhou Rong, Yongxin Hao, Ruixiang Zhou, Xunli Yin, Zhihua Shao, Lei Liang, Xueguang Qiao

**Affiliations:** Physics Department, Northwest University, No.229, Taibai Road (North), Xi’an 710069, China; qzrong2010@gmail.com (Q.R.); 2014112063@stumail.nwu.edu.cn (Y.H.); 1995828zhou@163.com (R.Z.); lixunyin@126.com (X.Y.); huahualovesnow@gmail.com (Z.S.); lianglei@opt.ac.cn (L.L.)

**Keywords:** fiber sensor, Fabry-Perot interferometer, UW imaging, seismic-physical-model

## Abstract

A fiber-optic Fabry-Perot interferometer (FPI) has been proposed and demonstrated for the ultrasound wave (UW) imaging of seismic-physical models. The sensor probe comprises a single mode fiber (SMF) that is inserted into a ceramic tube terminated by an ultra-thin gold film. The probe performs with an excellent UW sensitivity thanks to the nanolayer gold film, and thus is capable of detecting a weak UW in air medium. Furthermore, the compact sensor is a symmetrical structure so that it presents a good directionality in the UW detection. The spectral band-side filter technique is used for UW interrogation. After scanning the models using the sensing probe in air, the two-dimensional (2D) images of four physical models are reconstructed.

## 1. Introduction

The ultrasound wave (UW) detection is one of the critical ways for nondestructive testing on seismic physical models (SPM) which effectively bridges theory and field-scale experiments and allows us to obtain the changes of the acoustic response in the absence of a rock matrix and in a nearly ideal setting [1,2,3,4]. The detection of the UW field is traditionally realized by current-driven piezoelectric transducers (PZTs) [5,6,7]. However, this kind of transducer has several inherent drawbacks: the large size provides a poor spatial resolution; the production materials are sensitive to electromagnetic disturbances; the response frequency width is narrow owing to the resonance mechanism; and the sensitivity is small so PZTs must work under the support of the coupling agents, such as water and water-soluble polymer colloids. A considered solution is the optical mean that presents a different UW detection mechanism from PZT. Fiber-optic sensors, as a smart detection technology, have attracted considerable interest for their unique properties, and opened up a multitude of opportunities for single-point UW sensing in hard-to-reach electromagnetic spaces, with controllable cross-sensitivities and very compact size for the embedded measurement, as well as a good UW transmission line [8,9,10,11,12]. In order to realize the imaging of seismic physical model, a fiber sensor with a high UW sensitivity needs to be developed. When the simple spectral band-side filter technique is used for signal interrogation, the UW sensitivity is determined by the spectral slope [13,14]. The fiber Bragg grating (FBG) presents the single narrow resonance spectrum that is sensitive to the dynamic strain which has worked as a UW sensor in water medium [15]. Although further works are going on to improve the sensitivity of the FBG [16,17,18,19,20,21], the large Young’s modulus of the fiber materials and grating size makes the sensitivity promotion to some extent. To date, the FBG’s UW sensitivity is not large enough to realize imaging in air. In comparison, fiber-optic interferometers are more sensitive to the UW due to phase variation interrogation. Among the previous reports, the fiber-optic Fabry-Perot interferometer (FPI) has presented outstanding performances, as along with features such as compact size, flexible structure and high stability (low frequency vibration resistance) [22,23,24,25,26,27]. The UW sensitivity of the FPI is determined by the structure and material of the interference cavity. Specific optical films (thin silica [26], graphene [28], and parylene-C polymer [29]) have been used as sensing surfaces of FPIs for UW detection. However, they usually present low reflectivity (which results in a small signal-to-noise ratio (SNR) response) and complex fabrications, making them hard to apply in UW imaging in air. Therefore, most of the devices mentioned above also need to employ water as a propagation medium to decrease the UW power loss, and require a waterproof packaging technique to ensure the sensitivity and stability of the sensor for a long time in the water. For non-contact, two-dimensional (2D) SPM imaging in air, it is necessary to develop a directional UW sensor with an ultra-high sensitivity to collect UW signals containing the structure information of the scanned block. 

In this paper, we propose a FPI-based UW sensor and demonstrate it for SPM imaging in air. The sensor is simple, comprising just a single mode fiber (SMF) inserted into a ceramic tube with the end-face coated with an ultra-thin gold film. A spectral side-band filtering technique is used for UW interrogation at the wide frequency width. Finally, the 2D images of the SPM are achieved by reconstructing the detected UW signals using the time-of-flight approach. 

## 2. Sensor Fabrication and Sensing Mechanism

**Figure 1:**
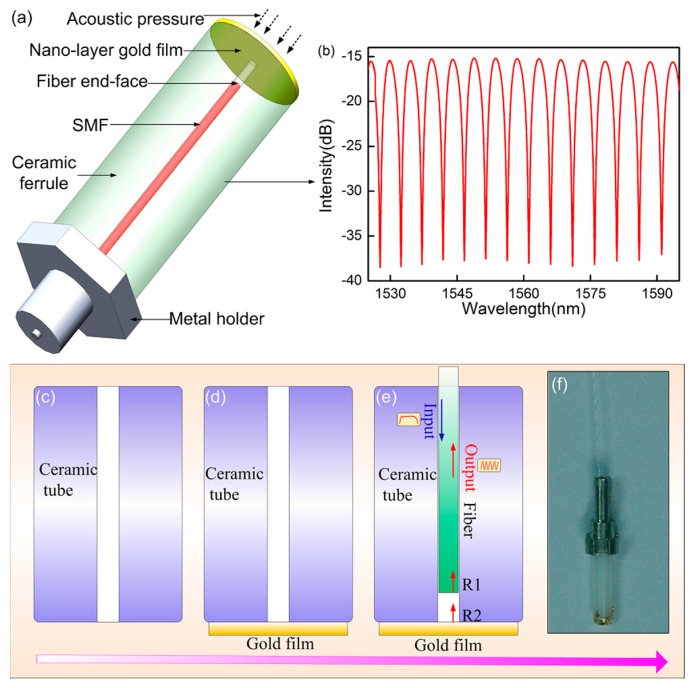
(**a**) Scheme diagram of UW sensor structure; (**b**) interference spectrum of sensor; (**c**–**e**) sensor fabrication process; (**f**) image of sensor before packaging protection.

Figure 1a demonstrates the structure and fabrication process of the sensor. It is seen that the end-face of the tube is first coated directly by a 130-nm-thick gold film. A leading-in SMF with a smooth end-face is inserted into the center hole of the ceramic tube. An air gap is formed by the two surfaces of the fiber and gold film, which can be adjusted by changing the insertion depth of the fiber. Once the confiscation is achieved, the input light will be reflected by the two surfaces (fiber end-face and gold film), and finally a well-defined interference spectrum is achieved with a fringe visibility of more than 25 dB (shown in Figure 1b) based on the FPI. Before bonding the gold film, the end-face of the ceramic tube must be clean enough to absorb the gold film closely by the Van Edward force among the molecules. Although the bonding force is weak, it is definitely strong enough to bond the gold film firmly because of its ultra-thin thickness of 130 nm. Compared to the sensor in the previous report [25], the proposed sensor in this work has several potential advantages: the higher reflectivity of the gold film promotes the light reflection, the better antioxidant capacity of the gold film makes the sensor work stably for a long time, and an improved packaging method makes the sensor more stable and sturdy for continuously scanning UW detection.

The sensing mechanism of the UW sensor is characterized theoretically by analyzing the interaction between the UW pressure and the optical interference. In this process, in order to simplify the discussion, we just consider the axial strain of the gold film induced by the UW pressure and ignore the shear stress because of the circular symmetric structure of the sensor and the nano-sized thickness of the gold film. When the UW is applied on the sensor in air, the acoustic pressure will deform the gold film. If the wavelength of the UW is much larger than the sensing region of the gold film, the UW can be regarded as a plane wave, and the UW pressure is represented as

(1) $$P(L,t)=P_{0}C\exp(j(\omega t-\frac{2\pi }{\lambda_{s}}L))$$

where *P*_0_ is the amplitude of the applied acoustic pressure, *C* is the coupling coefficient of the UW-to-gold film, ω is the angular frequency of the UW, *t* is the response time to the acoustic pressure of the sensor, *L* is the thickness of the gold film, and *λ_s_* is the wavelength of the UW in air.

As the UW propagates to the sensor, most of the power is reflected at the air-to-film interface owing to the large acoustic impedance difference between the optical fiber (18.9 kg/(m^3^·s)) and the air (0.0004 kg/(m^3^·s), resulting in a transmission coefficient close to 0 and a reflection coefficient close to 1. The acoustic pressure loading makes the film deform, resulting in the cavity length variation of the sensor. The strain of the sensor can be given by

(2) $$\xi (L,t)=\frac{P(L,t)}{E}$$

where *E* is the elastic modulus of the gold film. When the thickness of the nanolayer gold film is much smaller than the UW wavelength, the sensor structure’s influence on the UW transmission and acoustic pressure can be ignored. According to Equation (2), the cavity length change of the sensor, which is significantly determined by deformation of the gold film, is obtained by

(3) $$\Delta L=\Delta l=\xi \cdot L=\frac{P_{0}CL}{E}\exp\left(j(\omega t-\frac{2\pi }{\lambda_{s}}L)\right)$$

The analysis above presents that the sensor is a low-finesse FPI. In this case, as the laser is launched into the sensor, the power output of the sensor can be expressed as

(4) $$I(t)=2R\left(1-\cos\frac{4\pi nL(t)}{\lambda }\right)I_{i}$$

where *I_i_* is the incident light intensity, *R* is the reflectivity of the two surfaces of sensor, *n* is the refractive index of air, *L*(*t*) is the cavity length of the sensor, and *λ* is the laser wavelength.

According to Equation (4), if the sensor works in the quadrature phase bias point that is controlled precisely by adjusting the laser wavelength, the light intensity change with the cavity length change is derived as

(5) $$\begin{array}{ll} \Delta I & =\frac{8\pi nRI_{i}}{\lambda }\Delta L(t)=\frac{8\pi nRI_{i}}{\lambda }\Delta l(t)\\ & =\frac{8\pi nRI_{i}CL}{\lambda E}P_{0}\exp\left(j(\omega t-\frac{2\pi }{\lambda_{s}}L)\right) \end{array}$$

Equation (5) clearly shows that the output power of the sensor presents a high dependence on the UW pressure. Here the UW frequency is determined by the PZT source, and thus the output intensity of the sensor is determined by the amplitude of the UW pressure. In Equation (5), the item of $P_{0}\exp(j(\omega t-2\pi L/\lambda ))$ can be as simple as

(6) $$\frac{dI(t)}{dP(t)}=\frac{8\pi nRI_{i}CL}{\lambda E}$$

In practical applications, we should make a trade-off between the frequency range and sensitivity of the sensor by choosing suitable structure parameters, such as the sensor length and the thickness and diameters of the gold film.

## 3. Experimental Results and Discussion

**Figure 2:**
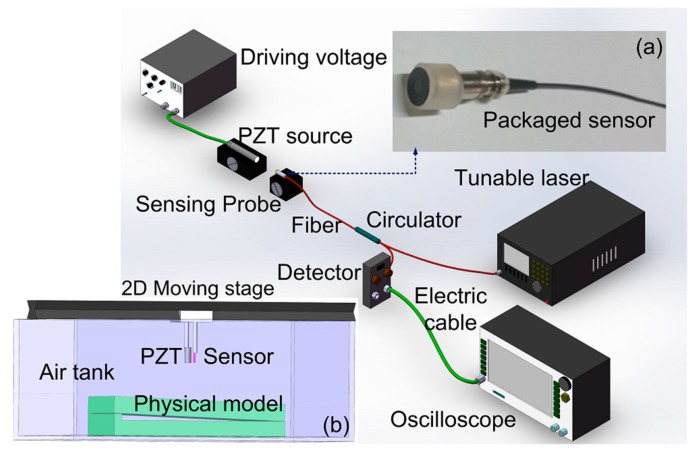
Schematic configuration of experiment setup for UW imaging, insets contain (**a**) photograph of sensor, and (**b**) schematic diagram of scanning imaging for physical models.

The schematic configuration of the UW sensing system is shown in Figure 2. A tunable laser (Santec, 710, Komaki, Aichi, Japan) with a 100 kHz linewidth and a 0.1 pm tunable resolution was employed as the light source and was launched into the sensing probe through a circulator. The reflection of the sensor was monitored by a photodiode (PD, New Focus, San Jose, CA, USA) with a bandwidth of 10 MHz at a 0 dB gain, and finally a signal was launched into an oscilloscope for signal analysis. The above sensing probe (shown in Figure 1f) was protected further to promote the firmness of sensor. The transmission SMF at the outside of the ceramic tube was inserted into a thin steel tube, and the end part of the sensor was protected by a plastic tube terminated by another firm sound-transparent film. This method protects the gold film well and does not influence the sensor’s response to ultrasonic waves. The two packaging processes are important to ensure the stability and sturdiness of the sensing probe during scanning images of the physical models. In order to characterize the sensor’s sensitivity in air, the sensor was held on a moving stage, and the PZT source, providing the UW pulse in the frequency range of 100 kHz to 10 MHz, was held on a fixed stage. The centers of the sensor and PZT were kept in the same line which was parallel to the experiment platform. The distance between the sensor and PZT was precisely controlled by the moving stage. In the following experiments, the sensor’s responses to the UW pulse of the different frequencies are demonstrated. 

**Figure 3:**
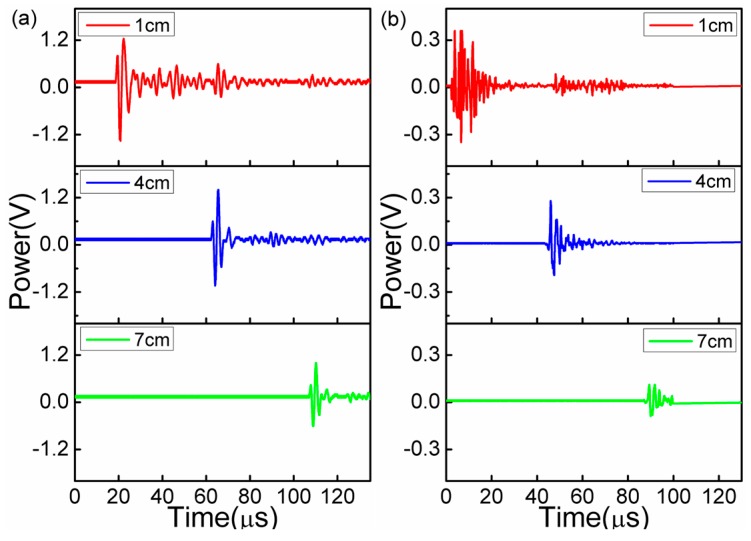
Time domain spectra of (**a**) 300 kHz and (**b**) 1 MHz UW versus increasing distances between PZT and sensor.

Figure 3a,b demonstrates the time domain spectra changes with the increasing distances at the fixed UW frequencies of 300 kHz and 1 MHz (which are usually used in SPM imaging), respectively. It is seen that the sensor presented high sensitivities to the two UW frequencies, which shows that the sensor is capable of measuring the UW of large frequency widths up to 1 MHz. As shown in Figure 3, with the increasing propagation distances, the detection voltage signal significantly decreased owing to the large loss of the UW energy in air. Given the noise voltage of 2 mV and the signal peak-to-peak voltage of 2.58 V (achieved in the red curve of Figure 3a), the signal-to-noise ratio of the sensor was calculated as 62.21 dB, which is larger than those of our previous works (27.96 dB for FBG-FP [30] and 24.08 dB for Micro-bubble FPI [31]). 

**Figure 4:**
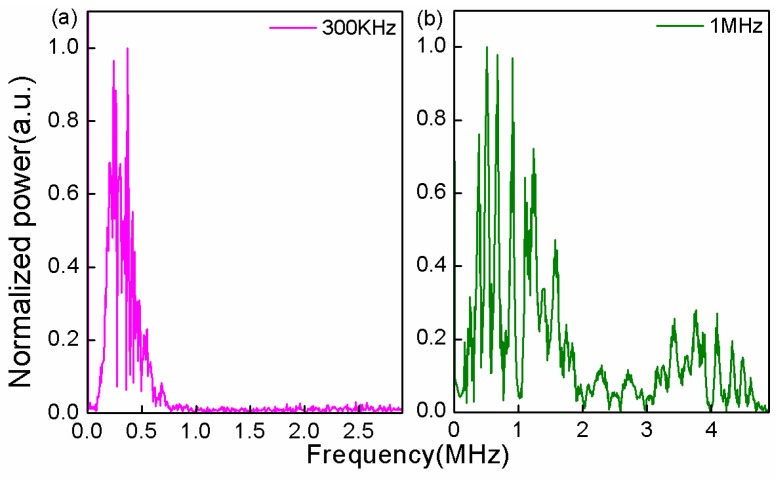
Frequency domain spectra of UW at the frequencies (**a**) 300 kHz; (**b**) 1 MHz.

Figure 4a,b is the frequency spectra that was achieved by the Fourier transform of the time domain spectrum. The main frequencies of 300 kHz and 1 MHz were in good agreement with the emission frequencies of the PZT source. In the frequency spectra, there were other resonance signals surrounding the main peaks of 300 kHz and 1 MHz, which were resulting from the PZTs that might launch multi resonances close to the main one. It further verifies that the sensor has a wide frequency band response to the UW components.

**Figure 5:**
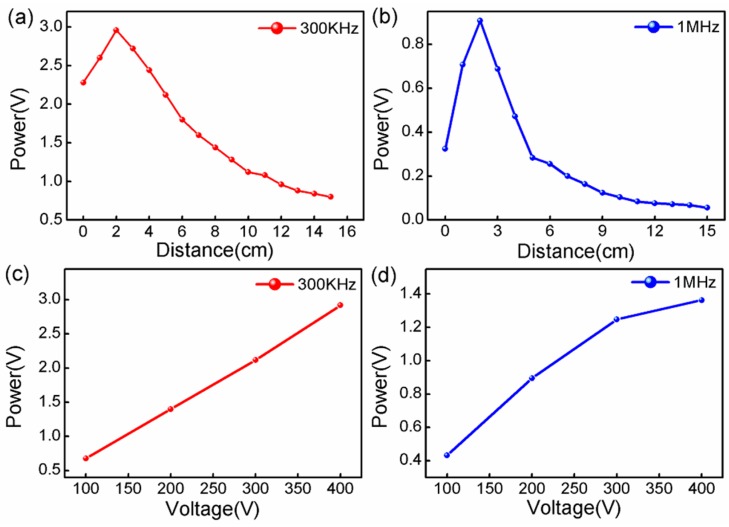
Experiment measurements: (**a**,**b**) UW power of 300 kHz and 1 MHz versus increasing distances at the fixed emission voltage; (**c**,**d**) UW power of 300 kHz and 1 MHz versus increasing voltage at a fixed distance.

In Figure 5a,b, we plot the peak-to-peak voltages of the detection signal as the function of the distances between the sensor and PZT. The signal intensity increased, then sharply decreased, which was attributed to the PZT used in the experiment being an acoustic focusing source. At the origin of several centimeters, the UW energy was focused on the sensor. When the separation was larger than the focusing length of the PZT, the UW energy decreased sharply owing to the UW diffusion and transmission loss. Figure 5c,d shows the peak-to-peak intensity of the detection signal (300 kHz and 1 MHz) under the driving voltage of 100 V to 400 V in the detection distance of 2 cm. As expected, the linear response function in Figure 5c was achieved, which is important for the dynamics range characterization and the UW imaging of the sensor. In Figure 5d, the appearance of the small nonlinear response of the sensor was mainly attributed to the high frequency UW power not increasing linearly with the driving voltage, which is totally determined by the PZT source.

**Figure 6:**
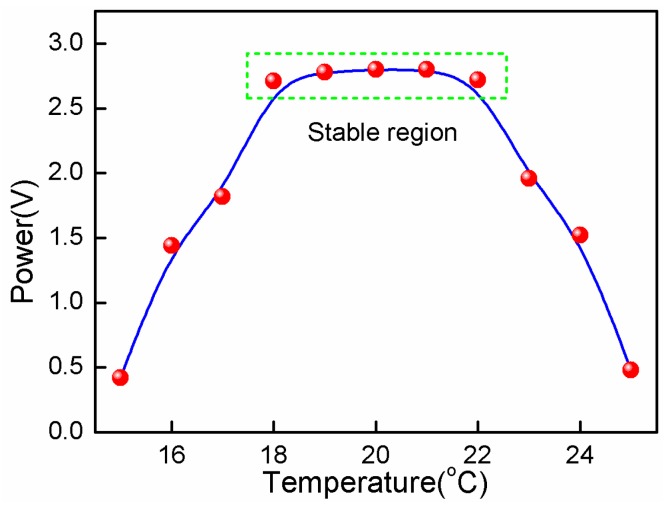
300 kHz UW power versus temperature change.

Temperature is a key perturbation factor for the detection stability, which makes the spectrum shift and the detection signal present low-frequency fluctuations. In order to characterize the temperature perturbation, the environment temperature surrounding the sensor increased from 20 °C to 26 °C, and decreased from 20 °C to 14 °C with a step of 1 °C, respectively. Figure 6 demonstrates the function of the signal intensity versus the temperature change. It was clearly seen that the sensor remained stable at the temperature range of ±2 °C near 20 °C. This anti-temperature range was enough for the stable imaging of the SPM which is usually kept in the experiment with a temperature perturbation of about ±1 °C.

**Figure 7:**
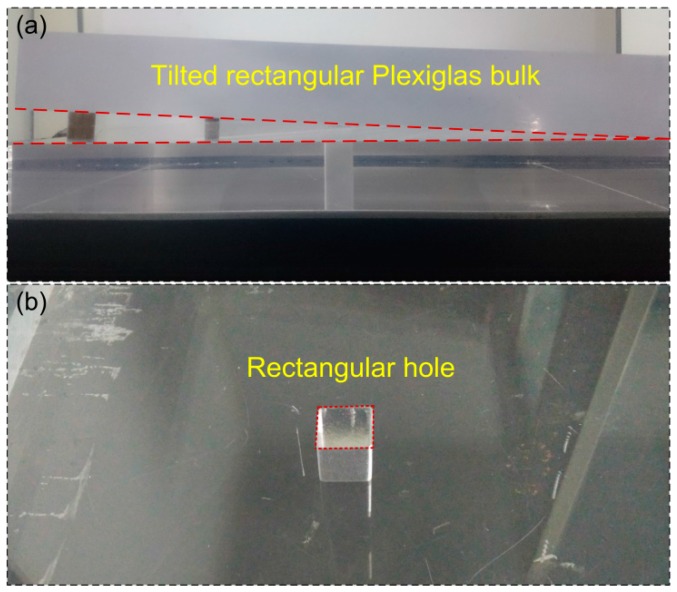
Photographs of two physical models, (**a**) tilt rectangular bulk; (**b**) rectangular hole in a bulk.

After the performance study of the sensor probe, the SPM imaging was demonstrated as follows. The experimental system of UW imaging of the SPM is shown in Figure 2. The tested models presented two shapes, as shown in Figure 7. One was a tilt rectangular Plexiglas bulk with a thickness of 5 cm and a length of 50 cm. The other one was a sunken model with a length and width of 3 cm and 2.5 cm in a larger rectangular Plexiglas block. The PZT source and the fiber sensor were held on an electric-driven stage with a spatial resolution of 2 μm for point-to-point scanning. The distance between the PZT and sensor was 3 cm. The air gap between the model and sensor was 5 cm. 

**Figure 8:**
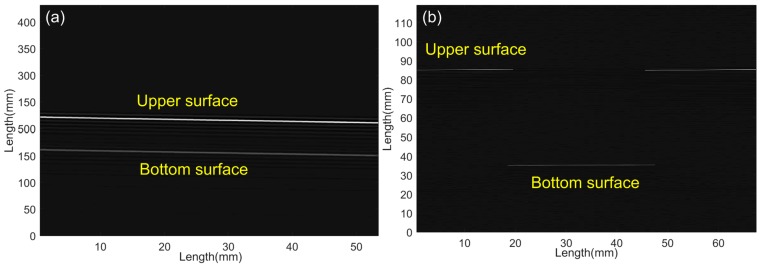
Images of physical models: (**a**) tilt rectangular bulk; (**b**) rectangular hole in a bulk.

Figure 8a,b shows the UW images of SPMs reconstructed by the time-of-flight approach. As expected, the images clearly show that, for the first model, two surfaces and the tilt angle of the model were achieved, and for second model, the edges of the rectangular sunken were also performed, which were in good agreement with the real model structures.

In our prior work [30], the UW imaging of SPMs was realized by the FBG-FP–based sensor. However, although UW-focused technology was employed to improve the performances of the sensor, the sensitivity was not high enough and the whole UW scanning process had to be finished under water. The key to success of the proposed sensor, detecting the UW in air, is its ultra-high sensitivity to the weak UW field, its large dynamic response range from 100 V to 400 V, and its wide-band frequency responses to 100 kHz and 1 MHz. The nanolayer gold diaphragm is a key component of the sensor, which is prone to sound pressure–induced slight deformation and provides high reflectivity (which guarantees the high SNR output). Further, the large dynamic range makes the sensor work stably at the environmental temperature fluctuation of ±2 °C. These excellent performances make the sensor a good candidate for the non-contact imaging of SPMs in air. As expected, the surfaces and boundaries of the two physical models were imaged clearly by reconstructing their reflecting UW signal. In our experiment, the models were placed in air which largely simplifies the imaging system, although the 300 kHz UW propagation presented a large loss owing to the acoustic impedance difference between the air and the models described above. In the experiment, the UW was partially reflected by the interface between the air and the models, i.e., the upper surfaces of the models. Meanwhile, the models of Plexiglas materials allowed the partial UWs to pass over, and then reflected them from the defects in models, especially by the bottom surfaces. According to the information of the UW transmission velocity in the air (340 m/s) and models (2700 m/s), and the time of flight shown in Figure 3b, the thickness and defect positions can be determined. By scanning the models and reconstructing the 2D image, the shape, size and inner structures were seen clearly, as shown in the above images. The high-precision imaging of the SPM was determined by two factors in this experiment. One was the sensor structure itself. During the scanning process, the compact size (with an effective sensing diameter of 125 μm) and the good directivity of the sensing structure made the sensor have a high space resolution, which was significantly important for the imaging of the small defects in the models. The other factor was the noise removal. It is necessary to employ a band-pass filter near 300 kHz for removing the unwanted noise frequencies coming from the surrounding electromagnetic interference and the PZT resonance. In addition, the digital filtering was employed to remove the other extra noise coming from the mode conversion in the physical models: the nonuniformity of the SPM induced the refraction and sound of speed variation in the model, and the reflections of other object surfaces (the bottom and sides of the water tank) also caused additional noise.

## 4. Conclusions

In this paper, we developed a fiber-optic FPI probe for non-contact UW imaging of SPMs in air. The interrogation of the sensor only requires one PD together with a tunable laser or side-band filter. The 2D images of four model blocks were reconstructed by the proposed UW scanning system. Compared with the PZT imaging device and hydrophone, the FPI probe provides a smaller structure and excellent performances of high sensitivity and spatial resolution. However, unfortunately, the exact frequency bandwidth of the sensor could not be characterized now because of the lack of the PZT sources and matching calibration microphone. Further improvement will be ongoing.
